# Experimental study on the treatment of norepinephrine transporter-overexpressing pheochromocytomas and paragangliomas: a synthetic lethality strategy combining ^131^I-MIBG with PARP inhibitors

**DOI:** 10.3389/fonc.2025.1681054

**Published:** 2025-10-29

**Authors:** Jieping Song, Lulu Zhang, Fan Qiu, Xiumin Shi, Rui Tian, Chuan Zhang, Xiaoyuan Li, Feng Wang

**Affiliations:** Department of Nuclear Medicine, Nanjing First Hospital, Nanjing Medical University, Nanjing, China

**Keywords:** pheochromocytoma, paraganglioma, ^131^i-mibg, PARPi, combination therapy

## Abstract

**Introduction:**

Pheochromocytomas and paragangliomas (PPGLs) are associated with poor prognosis especially in patients with metastatic spread. This study aims to investigate the therapeutic potential of ^131^I-MIBG and the PARP inhibitor fluzoparib monotherapies and their combination on two distinct PC12-derived stable cell lines: PC12-NET cells and PC12-NET-SDHB cells.

**Methods:**

Lentiviral transduction was used to generate PC12-NET cells overexpressing the norepinephrine transporter (NET) and PC12-NET-SDHB cells with suppressed succinate dehydrogenase subunit B (SDHB) expression. The specificity of PC12-NET cells to the ^131^I-MIBG was confirmed through desipramine inhibition assays. Subsequently, the synergistic effects of ^131^I-MIBG and fluzoparib monotherapies and their combination were assessed *in vitro* through proliferation assays, cell cycle analysis and apoptosis analysis in both cell lines.

**Results:**

NET overexpression significantly enhanced ^131^I-MIBG uptake in PC12-NET cells, confirming NET expression as a critical determining of ^131^I-MIBG therapeutic efficacy. The combination of ^131^I-MIBG with fluzoparib exhibited substantial synergistic effects in PC12-NET cells, leading to a significant G2/M phase arrest and a marked increase in apoptosis compared to monotherapy, particularly where monotherapy alone was ineffective. Notably, although low expression of the SDHB did not alter cell proliferation in response to ^131^I-MIBG treatment, PC12-NET-SDHB cells exhibited a greater sensitivity to fluzoparib-induced G2/M phase arrest than PC12-NET cells.

**Discussion:**

The combined of ^131^I-MIBG with PARP inhibitor demonstrated a synergistic antitumor effect in PC12-NET cells. While PC12-NET-SDHB cells display comparable sensitivity to ^131^I-MIBG as PC12-NET cells, they exhibited heightened responsiveness to PARP inhibitor treatment.

## 1 Introduction

Pheochromocytomas and paragangliomas (PPGLs) are neuroendocrine tumors that arise from chromaffin cells, with pheochromocytomas develop within the adrenal medulla while paragangliomas occur in extra-adrenal locations (1). Both tumor types are frequently associated with catecholamine hypersecretion, which drives their clinical presentation (1). Rather than being classified as simply benign or malignant, PPGLs exhibit a variable biological behavior, with metastases develop in approximately 5–26% of cases (2). Prognosis is highly heterogeneous, with mortality rates of 37% over ten years and 29% over five years (3). These findings highlight the critical need for effective treatment options for metastatic PPGLs.

The norepinephrine transporter (NET) is a critical membrane protein that plays a vital role in the reuptake and recycling of norepinephrine in synaptic clefts and adrenal chromaffin cells (4). Recent research has increasingly focused on its role in mediating monoamine uptake and storage mechanisms, particularly in PPGLs and neuroblastoma (5). Beyond serving as a molecular target for tumor diagnosis, NET also acts as a key mediator for radiotherapeutic agents such as ¹³¹I-metaiodobenzylguanidine (¹³¹I-MIBG), which utilizes its substrate specificity for targeted treatment (6). For patients with metastatic PPGLs exhibiting high MIBG uptake on scintigraphy, targeted radionuclide therapy with ^131^I-MIBG presents a viable theranostic option (7, 8). However, treatment efficacy is often limited in case with germline mutations in the succinate dehydrogenase subunit B (SDHB). These SDHB-mutant PPGLs frequently demonstrate inherent or acquired resistance to ^131^I-MIBG, leading to suboptimal treatment responses (1, 9). The mechanisms underlying this resistance are complex, involving dysregulated intracellular signaling pathways and perturbations in cellular survival networks that collectively impair drug uptake and retention (10, 11). Consequently, despite available treatment such as ^131^I-MIBG, the management of metastatic PPGLs remains a considerable challenge.

Poly(ADP-ribose) polymerase (PARP) is an essential enzyme in the base excision repair (BER) pathway and plays a particularly critical role in tumors with homologous recombination (HR) deficiency (12, 13). PARP inhibitors (PARPi) utilize the principle of synthetic lethality to enhance tumor sensitivity to radiotherapy and chemotherapy, establishing themselves as promising agents in oncology. These agents have shown significant clinical efficacy in BRCA-mutated tumors, including ovarian and breast cancers (14, 15). Growing evidence indicates that most PPGLs are genetically driven and can be categorized into three molecular subtypes: pseudohypoxic, kinase- signaling, and Wnt- signaling. SDHB mutations, which is classified under the pseudohypoxic subtype, disrupt the tricarboxylic acid (TCA) cycle and impair catecholamine metabolism. These metabolic alterations often result in mild initial symptoms, leading to frequent diagnosis at advanced stages. Specifically, SDHB loss compromises succinate dehydrogenase activity, causing a loss of electrons produced by mitochondrial complex II and subsequent dysregulation of upstream metabolic pathways. This metabolic alteration enhances the activity of α-ketoglutarate dehydrogenase, promoting the oxidation of NADH to NAD+ (16). The subsequent rise in NAD+ levels stimulate the NAD+-dependent PARP/BER DNA repair pathway, thereby augmenting the capacity of tumor cells to repair DNA damage. This metabolic disturbance triggers hyperactivation of the NAD+-dependent PARP-mediated BER pathway, which further exacerbates treatment resistance (17–19). Therefore, we propose that PARPi may effectively inhibit the upregulated PARP/BER repair mechanisms in tumors harboring SDHB mutations, thereby enhancing the sensitivity of these patients to conventional therapy and representing a promising therapeutic strategy for metastatic PPGLs (20, 21).

This study aimed to develop novel strategies to overcome therapeutic resistance in metastatic PPGLs. We established cellular models with high NET expression and low SDHB expression to evaluated the responses to ^131^I-MIBG and fluzoparib, both as monotherapies and in combination. Our goals were to elucidate the influence of NET and SDHB expression on therapeutic sensitivity and to assess whether combining ^131^I−MIBG with PARPi could effectively overcome resistance, especially in cells with low expression of SDHB. These findings provide critical insights for optimizing treatment strategies and support the clinical translation of this combination therapy.

## 2 Materials and methods

### 2.1 Cell culture

PC12 rat adrenal chromaffin tumor cells were purchased from Shanghai Wenren Biotechnology Co., Ltd (Shanghai, China). The cells were cultured in RPMI-1640 medium (Gibco, USA) supplemented with 10% heat-inactivated horse serum (NEWZERUM, New Zealand), 5% fetal bovine serum (FBS, Gibco, USA), and 1% penicillin/streptomycin (Gibco, USA) in a 37°C, 5% CO_2_ and humid atmosphere.

### 2.2 RNA interference

Lentiviral particle encoding short hairpin RNA (shRNA) targeting *Slc6a2* and *SDHB* were purchased from Shanghai Genechem Co., Ltd. The target sequence for *SDHB* shRNA was 5’-GCAGTTCTCATGCAGGCTTAT-3’. PC12 cells in the logarithmic growth phase were digested by trypsin and resuspended with a complete medium at a density of 40000–60000 cells/ml. The cells were inoculated into the culture plate to ensure that the plate amount reached about 20-30% when infected. Within 72 hours post-infection, the medium was replaced with fresh complete medium containing 4 μg/mL puromycin (MedChemExpress, USA) for 48 hours to select transduced cells. Consequently, two stable cell lines were established including PC12-NET with high NET expression and PC12-NC1 serving as a negative control. Subsequently, PC12-NET cells were then further transduced with a lentiviral vector encoding *SDHB* shRNA. Following selection under 1000 μg/mL Geneticin (G418 sulfate, APExBIO, USA), two additional cell lines were established: PC12-NET-SDHB cells with stable *SDHB* knockdown and PC12-NC2 serving as the corresponding negative control.

### 2.3 RNA extraction and quantitative real-time PCR

The Quantitative PCR primers for *NET-F/R、SDHB-F/R、beta-Actin-F/R* were synthesized by GenScript Biotech Co., Ltd (Nanjing, China). Total RNA was extracted from PC12 cells by *SteadyPure* Universal RNA Extraction Kit (Accurate Biology, China) according to the manufacturer’s instructions. The concentration and purity of extracted RNA samples were detected by Nanodrop 2000C spectrometer (Thermo Scientific, USA). Then, total RNA was reversely transcribed to cDNA with a Evo M-MLV reverse transcription Kit (Accurate Biology, China). qPCR was then performed on QuantstudioTM Dx Real‐Time PCR System (Thermo Fisher Scientific, USA) by using SYBR^®^ Green *Pro Taq* HS qPCR Kit (Agbio, China). The program was run with the following settings: preheated at 95°C for 30 s, denatured at 95°C for 5 s, annealed at 60°C for 30 s. The total of 40 cycles were repeated. The Cycle threshold (Ct) values of the target genes were corrected by using the internal reference *beta-Actin* of the same sample. Primer sequences used in this study are shown in **Table 1**.

**Table 1:** Primers used for qPCR.

Gene	Primer sequences(5’to3’)	
*Slc6a2*	Forward	TAAGAAGTCAGGTCCAGCACC
	Reverse	AGTAGAGCAAGGAAGGCACC
*SDHB*	Forward	CAGAGTCGGCCTGCAGTTTC
	Reverse	TAGAGCATCCAGCACCATCG
*beta-Actin*	Forward	CAGAGCAAGAGAGGCATCCT
	Reverse	GTCATCTTTTCACGGTTGGC

### 2.4 Western blotting assay

Total protein was isolated from cells with the Total Protein Extraction Kit (KGB5202-100, Keygen Biotech), and concentrations were measured using a BCA assay (KGB2101-5000, Keygen Biotech). The lysates were separated by SDS‐PAGE and transferred to polyvinylidene difluoride (PVDF) membranes. After blocking with 5% BSA for 2 hours, the membranes were incubated overnight at 4°C with primary antibodies against NET and SDHB. The following day, they were probed with an HRP-conjugated anti-rabbit IgG antibody (1:5000, AB0101, Abways) for 1 hour at 25°C. Protein bands were visualized using chemiluminescence detection reagents (ChemiScope 6100BZ; CLINX).

### 2.5 Cell uptake and blocking assays

#### 2.5.1 Cell uptake assay

^131^I-MIBG was purchased from Atomic High-Tech Co., Ltd (Beijing, China). PC12-NET cells in logarithmic growth phase were inoculated into 24-well plates and incubated them for 18 h. The adherent cells were co-incubated with ¹³¹I-MIBG (2 μCi/well) and ¹³¹I-NaI (2 μCi/well) for 24 h and 48 h. After incubation, the medium was removed and the cells were washed with cold PBS three times. Then, 300 μL of 0.2 M NaOH was added, and the cell lysate was collected into 1.5 mL Eppendorf tubes. Radioactivity in each tube was measured with an auto-gamma counter (PerkinElmer WIZARD2 2480, MA, USA) and AD% values were calculated. Protein content was measured using the BCA method. The AD% values were normalized to protein content to calculate the AD%/g value, which was used to assess the uptake ability of cells for ¹³¹I-MIBG and ¹³¹I-NaI. Additionally, PC12 and PC12-NET cells in logarithmic growth phase were plated in 24-well plates and co-incubated with ¹³¹I-MIBG (2 μCi/well) at different time points to calculate AD% values and assess the uptake ability of both cell groups for ¹³¹I-MIBG.


AD%=Radioactive count of the sample÷Radioactive count of the standard substance

AD%/g=AD%÷Protein content

#### 2.5.2 Blocking assay

PC12-NET、PC12-NC1 and PC12 cells in logarithmic growth phase were inoculated into 24-well plates and incubated them for 18 h. The adherent cells were co-incubated separately with the NET-specific inhibitor desipramine (DMI, MedChemExpress, USA) for 30 minutes before administering ¹³¹I-MIBG (2 μCi/well) for 24 h. The AD%/g value was calculated to assess the impact of DMI on ¹³¹I-MIBG uptake.

### 2.6 CCK-8 detection of cell proliferation activity

PC12, PC12-NET and PC12-NET-SDHB cells in logarithmic phase were inoculated into 96-well plates and treated with different concentration gradients of ^131^I-MIBG, fluzoparib, and combination therapy. Drugs were administered according to the experimental design, and the medium was changed at indicated time points after treatment. The CCK-8 kit was used to detect cell viability. CCK-8 reagent (10 μL/well) were mixed with the medium, then PC12, PC12-NET, and PC12-NET-SDHB cells were incubated at 37°C in a 5% CO_2_ incubator for 1 hour. The Optical density (OD) at 450 nm was thereafter examined for cell count and viability using PHomo microplate reader (Anthos Labtec Instruments GmbH, Austria). The cell survival rate (%) was calculated as follows: Cell survival rate (%) = (cell OD value − background OD value) ÷ (control cell OD value − background OD value) × 100%. The IC_50_ value of the drug was calculated to evaluate the inhibitory effect of drug treatment on cell proliferation.

### 2.7 Cell cycle experiment

PC12-NET and PC12-NET-SDHB cells in logarithmic phase were inoculated into 6-well plates and treated with different concentration gradients of ^131^I-MIBG, fluzoparib, and combination therapy. Drugs were administered according to the experimental design, and the medium was changed at indicated time points after treatment. After drug incubation, cells were harvested and fixed in 1 mL of 70% ice-cold ethanol and stored at -20°C until used. Next, the fixed cells were washed with PBS solution twice and incubated with RNase at 37°C for 30 min. Finally, the cells were stained with propidium iodide (PI), and analyzed for cell cycle distribution using flow cytometry. In part of the experiments, the Sub-G1 peak was specifically selected to assess the response of cells to drugs based on the degree of DNA fragmentation. Analysis was performed using the cell cycle analysis module included in Kaluza software, and results were exported for statistical analysis using GraphPad Prism software (version 6.0).

### 2.8 Cell apoptosis detection

PC12-NET cells in logarithmic phase were inoculated into 6-well plates to ensure that the plate amount reached about 70% when drugs were administered. Cells were co-incubated with ^131^I-MIBG for 24 h and fluzoparib for 48 h to assess the effects of monotherapy and combination therapy on apoptosis. Double staining with Annexin V-FITC and PI was performed, and flow cytometry was utilized to detect apoptosis, supplemented by fluorescence microscopy to observe cell morphology and evaluate drug-induced apoptosis.

### 2.9 Statistical analysis

Statistical analysis was performed using GraphPad Prism (version 6.0), and Statistical Package for the Social Sciences (version 20.0) software. Statistical data conforming to a normal distribution are expressed as mean ± standard error (mean ± SE). Independent sample t-tests were utilized to analyze intergroup differences. A p ≤ 0.05 was considered statistically significant.

## 3 Results

### 3.1 Establishment of NET- overexpression and SDHB- knockdown PC12 cell lines

**Figure 1:**
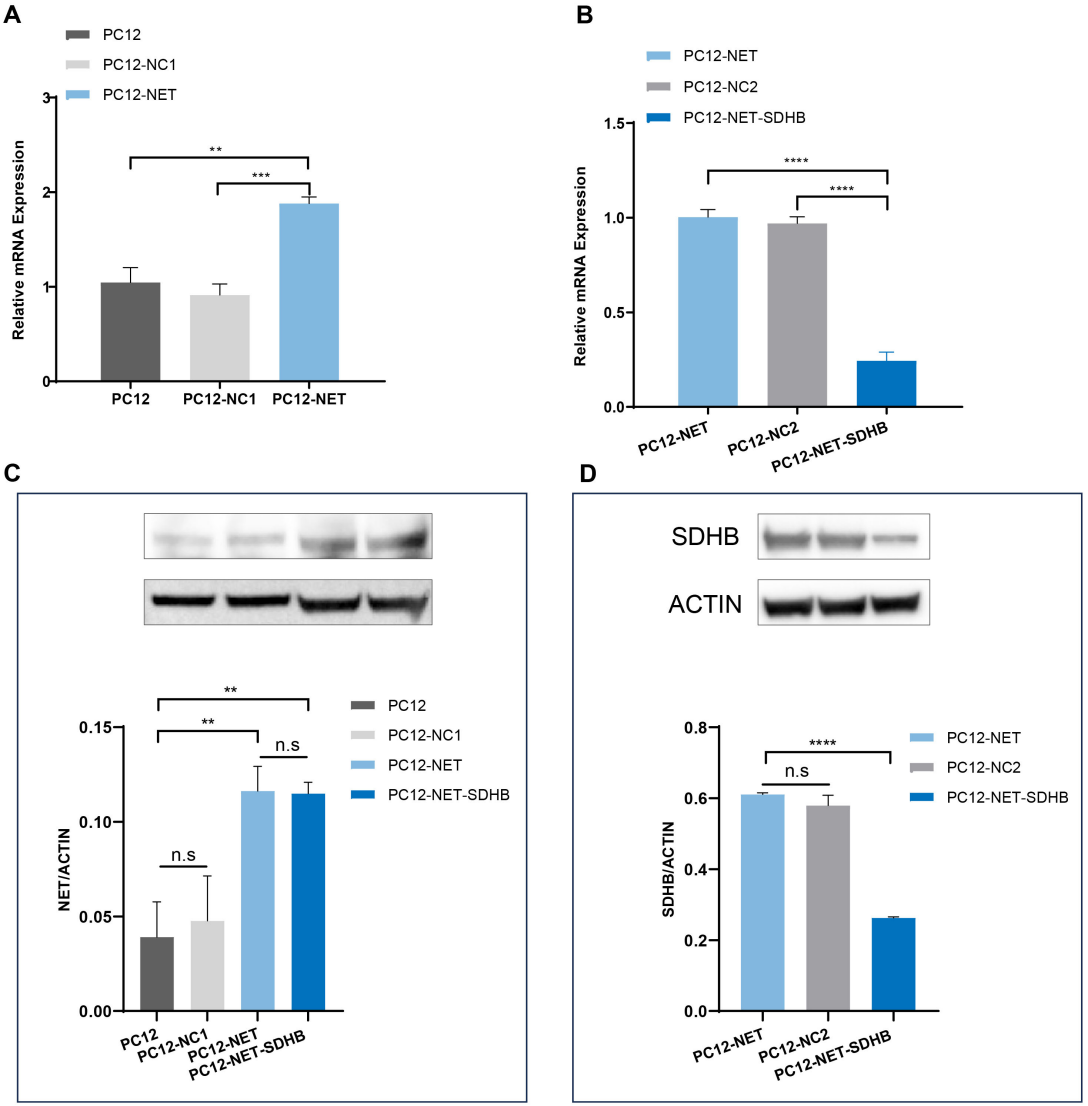
Analysis of NET and SDHB expression by qPCR and Western blot. **(A)** The qPCR analysis of NET (*Slc6a2*) mRNA expression in PC12, PC12-NC1, PC12-NET cells. **(B)** The qPCR analysis of *SDHB* mRNA expression in PC12-NET, PC12-NC2, PC12-NET-SDHB cells. **(C)** Western blot analysis of NET protein expression in the corresponding cell lines. β-Actin was used as a loading control. **(D)** Western blot analysis of SDHB protein expression in the corresponding cell lines. β-Actin was used as a loading control. **p < 0.01, ***p < 0.001, ****p < 0.0001, n.s: no significance.

PC12 cells were infected with lentivirus vector carrying the *Slc6a2* (NET) gene, and the stable cell line PC12-NET was selected using puromycin. The qPCR analysis revealed that NET mRNA expression in PC12-NET cells was significantly increased by 1.88-fold compared to wild-type PC12 cells (p = 0.0013), and by 2.06-fold compared to the negative control PC12-NC1 cells (p = 0.0001) (**Figure 1A**). Western blot analysis further confirmed the successful overexpression at the protein level, showing a consistent increase in NET protein expression compared to both control lines (**Figure 1C**).

Subsequently, PC12-NET cells were infected with a lentivirus carrying *SDHB* shRNA, and the stable cell line PC12-NET-SDHB was obtained through Geneticin (G418 Sulfate) (1000 μg/mL) selection. The qPCR analysis demonstrated a pronounced reduction in *SDHB* expression in PC12-NET-SDHB cells, with decrease of 75.58% compared to PC12-NET cells (p < 0.0001) and 74.82% compared to the negative control PC12-NC2 cell (p < 0.0001) (**Figure 1B**), demonstrating effective knockdown in the PC12-NET-SDHB group. This knockdown was further validated by Western blot, which showed a significant decrease in SDHB protein expression in PC12-NET-SDHB cells compared to the control groups (**Figure 1D**).

### 3.2 Cell uptake and blocking results

**Figure 2:**
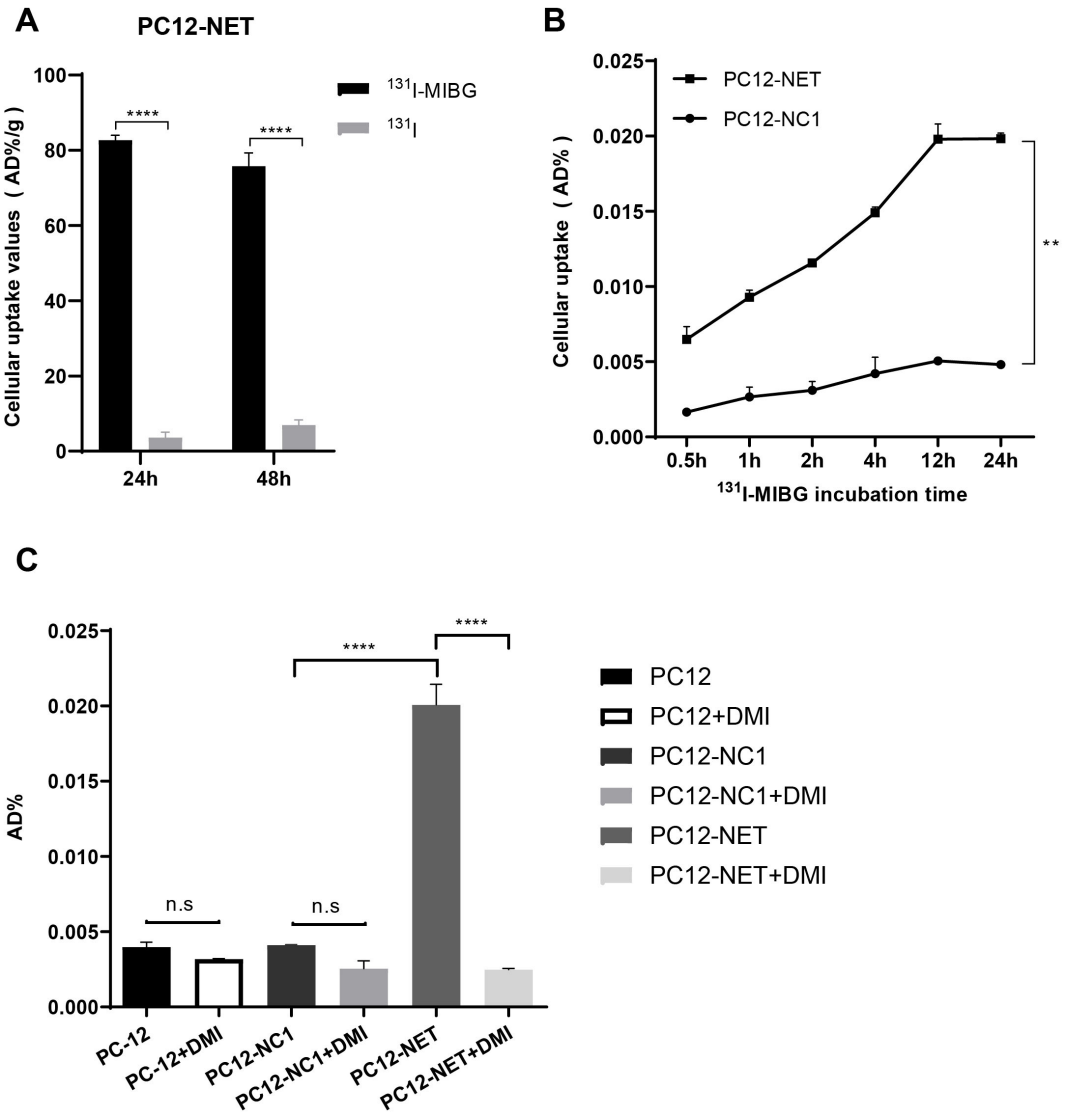
Cell uptake and blocking assays of ^131^I-MIBG. **(A)** Radioactive uptake of ^131^I-MIBG and free ^131^I^-^ in PC12-NET cells after 24 and 48 h of incubation. **(B)** Time-dependent uptake of ¹³¹I-MIBG in PC12-NET and negative control PC12-NC1 cells. **(C)** Uptake of ¹³¹I-MIBG in PC12, PC12-NC1, and PC12-NET cells with or without pre-treatment with the NET inhibitor DMI. **p < 0.01, ****p < 0.0001, n.s: no significance.

The radioactive uptake of ^131^I-MIBG and ^131^I^-^ in PC12-NET cells was evaluated after 24 and 48 h of coincubation (**Figure 2A**). After 24 h of coincubation, the cellular uptake of ^131^I-MIBG was 82.67 ± 1.32 AD%/g, significantly higher than that of ¹³¹I^−^ for 3.59 ± 1.47 AD%/g (p < 0.0001). A similar trend was observed at 48 h, the uptake values were 75.80 ± 3.48 AD%/g for ^131^I-MIBG and 6.91 ± 1.47 AD%/g for ¹³¹I^−^ (p < 0.0001). These results revealed that the targeted delivery of MIBG relies primarily on NET-mediated active transport rather than passive diffusion or nonspecific endocytosis.

To further validate NET-specific uptake, ¹³¹I-MIBG accumulation was compared between PC12-NET and PC12-NC1 cells at different time points (**Figure 2B**). The uptake of ^131^I-MIBG in PC12-NET cells increased in a time-dependent manner, whereas no significant change was observed in PC12-NC1 cells. A paired t-test exhibited a statistically significant difference between PC12-NET and PC12-NC1 cells across time points (p = 0.002). These findings indicate that NET expression is essential for the time-dependent accumulation of ^131^I-MIBG and confirm the reliability of PC12-NC1 as a negative control, ruling out the influence of the viral vector on the transport system.

To assess the specificity of ¹³¹I-MIBG uptake, PC12, PC12-NC1, and PC12-NET cells were divided into uptake and blocking groups. Cells in the blocking group were pre-incubated with DMI for 30 min prior to the addition of ¹³¹I-MIBG. All cells were then co-incubated with ^131^I-MIBG for 24 h, after which radioactive uptake were measured (**Figure 2C**). The radioactive uptake of ^131^I-MIBG in PC12-NET cells was significant higher in uptake group (0.02007 ± 0.00079 AD%/g) compared to that of DMI-block group (0.00248 ± 0.00005 AD%/g; p < 0.0001). In contrast, no significant differences were observed between two groups in either wild-type PC12 or PC12-NC1 cells. Furthermore, the radioactive uptake in DMI-blocked PC12-NET cells did not differ significantly from that in either PC12 or PC12-NC1 cells. These results revealed that NET is the specific pathway responsible for ^131^I-MIBG uptake, thereby supporting the mechanistic basis for ^131^I-MIBG as a targeted NET therapeutic agent.

### 3.3 Effects of ^131^I-MIBG and fluzoparib on cell proliferation in PC12-NET cells

**Figure 3:**
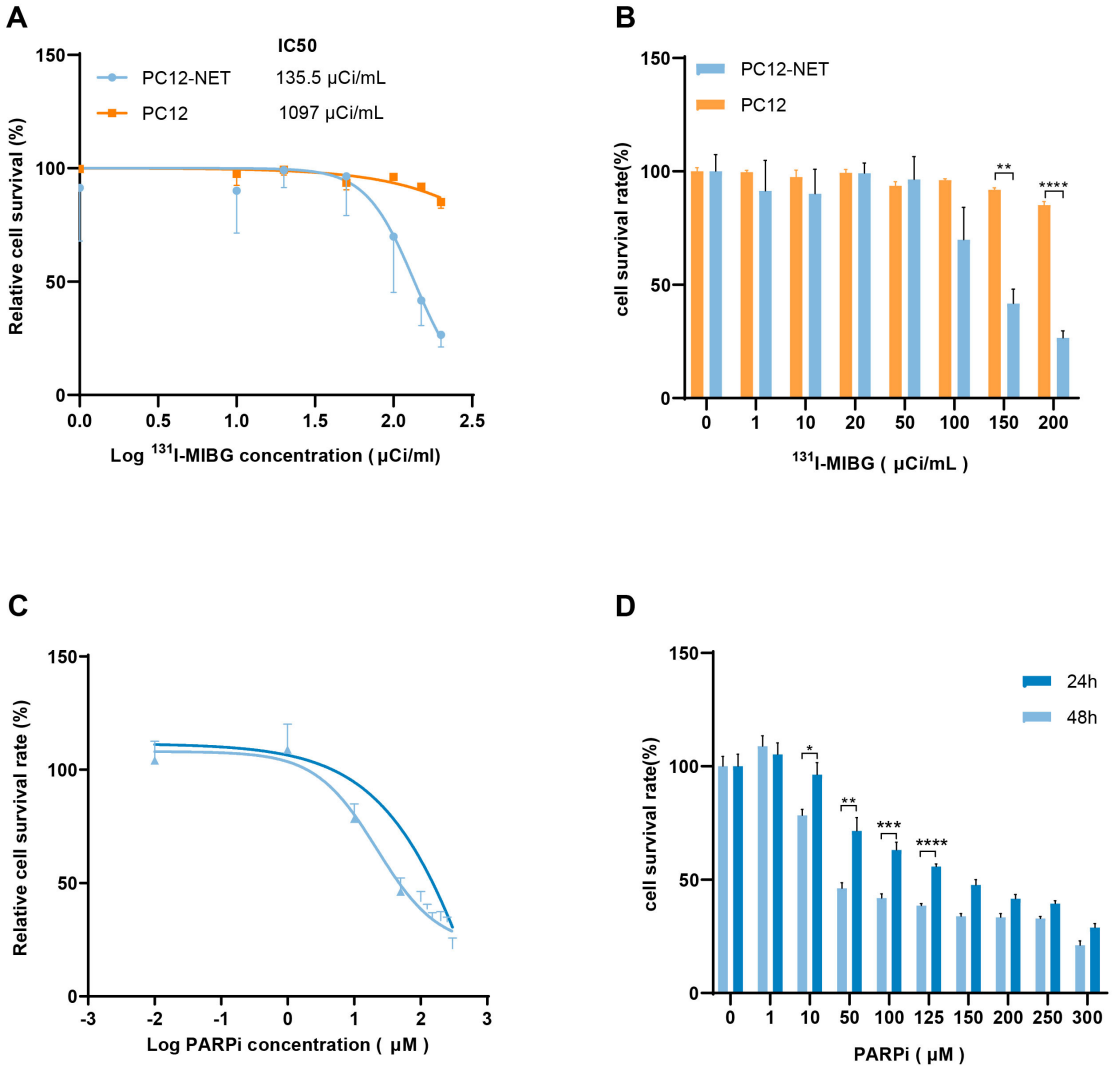
Effects of ^131^I-MIBG and fluzoparib on PC12-NET cells proliferation. **(A, B)** Survival curves and bar chart of PC12-NET and wild-type PC12 cells after treatment with increasing doses of ^131^I-MIBG. **(C, D)** Survival curves and bar chart of PC12-NET cells after treatment with increasing doses of fluzoparib for 24 and 48 h. *p < 0.05, **p < 0.01, ***p < 0.001, ****p < 0.0001.

PC12-NET and PC12 cells were incubated with increasing doses of ¹³¹I-MIBG for 24 h. After drug removal, cells were subsequently cultured in normal medium for an additional 24 h, and cell proliferation was assessed using the CCK-8 assay. ^131^I-MIBG significantly inhibited PC12-NET cell proliferation dose-dependently, with cell proliferation rates of 41.7% ± 6.4% at 150 μCi/mL and 26.6% ± 3.1% at 200 μCi/mL (**Figure 3A**). Compared to the 0 kBq/mL control group, the decrease in cell proliferation rates for both groups was statistically significant (p = 0.006, p = 0.007, respectively). The half-maximal inhibitory concentration (IC_50_) was calculated to be 135.5 μCi/mL. In contrast, PC12 cells were insensitive to ^131^I-MIBG treatment, indicating a decrease in cell proliferation solely at high doses(IC_50_ = 1097 μCi/mL), presumably attributed to the direct cytotoxic effect of beta radiation rather than NET-mediated uptake (**Figure 3A**). A comparison of proliferation rates between PC12-NET and PC12 cells after treatment with 150 and 200 μCi/mL of ^131^I-MIBG demonstrated a statistically significant difference between these two groups (p = 0.0014, p < 0.0001, respectively) (**Figure 3B**). These results demonstrate that NET expression is a key factor in determining ^131^I-MIBG efficacy. Cells with high NET expression can actively transport the drug, achieving efficient killing at low doses. Conversely, PC12 cells demonstrated proliferation inhibition at extremely high doses (>1000 μCi/mL) without dose dependence, indicating that ^131^I-MIBG is not significantly toxic to NET-negative cells at standard doses.

The effect of fluzoparib on cell proliferation in PC12-NET cells was further evaluated at varying concentrations and incubation times (**Figures 3C, D**). After 24 h of treatment, the cell survival rates were 96.3% ± 11.7% at 10 μM and 63.1% ± 7.6% at h 100 μM, with an IC_50_ of 146.8 μM. Extending treatment to 48 h, the cell survival rates reduced to 78.3% ± 6.0% at 10 μM and 41.9% ± 4.0% at 100 μM, with an IC_50_ of 62.71 μM. The 2.3-fold decrease in IC_50_ with prolonged exposure h demonstrates that fluzoparib exerts time-dependent cytotoxicity, which is consistent with the progressive accumulation of DNA damage mechanism of PARPi.

### 3.4 Effects of treatment on the cell cycle in PC12-NET cells

**Figure 4:**
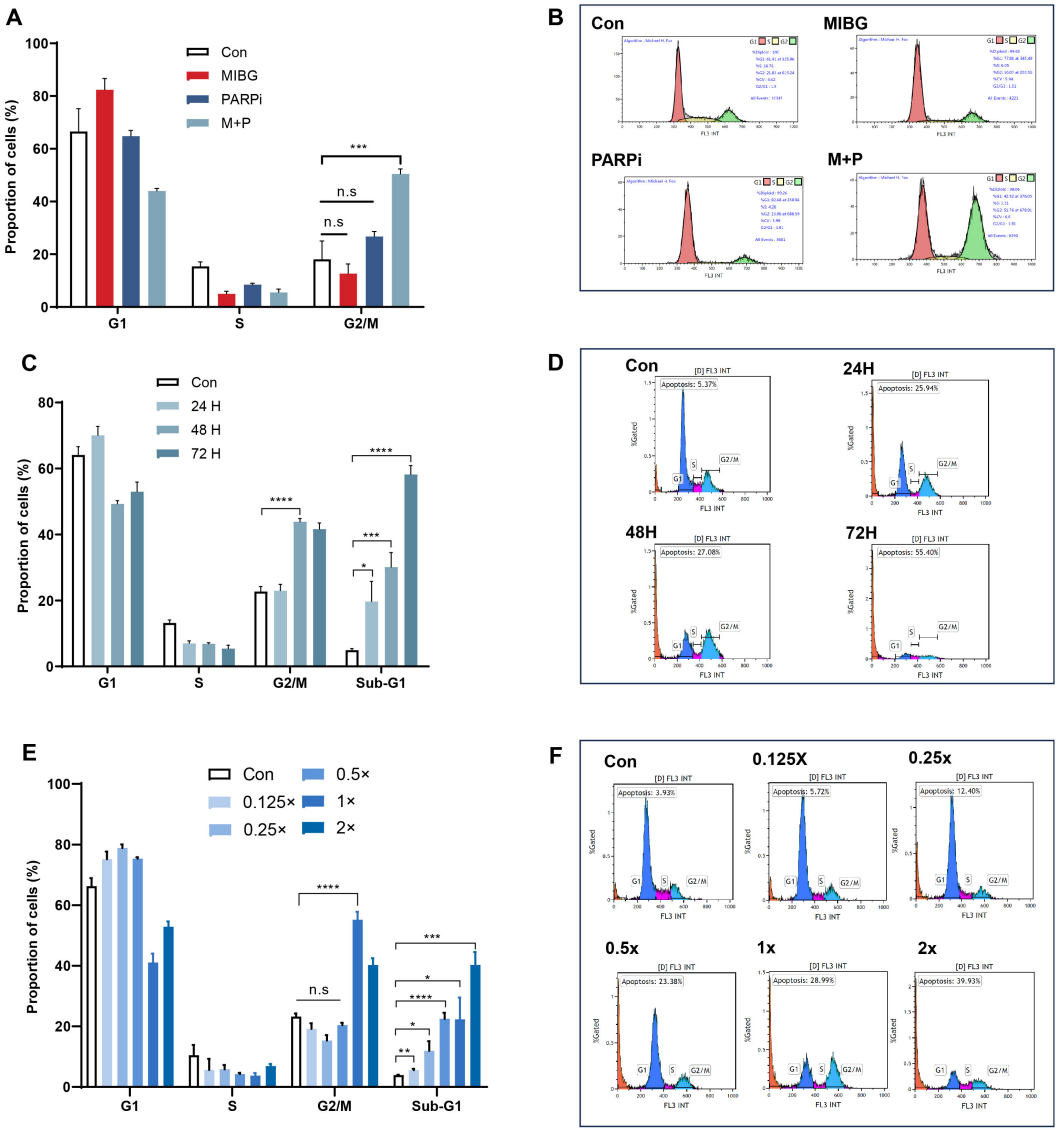
Effects of ^131^I-MIBG and fluzoparib treatments on the cell cycles phases in PC12-NET cells. **(A, B)** Quantitative analysis and distribution of cell cycle phases in control and treatment groups. **(C, D)** Quantitative analysis and distribution of cell cycle phases in control and treatment groups after different incubation times with fluzoparib. **(E, F)** Quantitative analysis and distribution of cell cycle phases in groups treated with varying doses of the combination therapy. *p < 0.05, **p < 0.01, ***p < 0.001, ****p < 0.0001, n.s: no significance.

PC12-NET cells were grouped and treated with low-dose monotherapy using 100 μCi/mL ^131^I-MIBG (MIBG group) or 10 μM fluzoparib (PARPi group), and combination therapy (M + P group). The combination therapy protocol consisted of 24 h incubation with ¹³¹I−MIBG followed by medium replacement and a further 24 h treatment with fluzoparib. The results revealed that only the combination therapy caused G2/M phase arrest, increase the proportion of G2/M phase cells from 18.1% ± 4.0% in the control group to 50.5% ± 1.1% (p = 0.001). Conversely, neither ^131^I-MIBG or fluzoparib monotherapy group and exhibited significant G2/M phase arrest (p = 0.30, p = 0.11, respectively) (**Figures 4A, B**). These results revealed that neither treatment alone (100 μCi/mL MIBG or 10 μM fluzoparib) reached an effective dose threshold (consistent with previous IC_50_ values), whereas the combination therapy exhibited a significant therapeutic effect.

To further optimize the combination treatment, we evaluated the effect of varying fluzoparib exposure durations on cell cycle. After incubating with ^131^I-MIBG (100 μCi/mL) for 24 h, the PC12-NET cells were then treated with fluzoparib (10 μM) for 24, 48, and 72 h. The 48 h fluzoparib treatment produced the most significant G2/M phase arrest, with the proportion of G2/M phase cells increasing from 22.7% ± 0.9% to 43.9% ± 0.6% (p < 0.001). Furthermore, a time-dependent increase in the Sub-G1 population was observed, which indicate apoptotic cells. The proportion of Sub-G1 phase cells gradually increased from 5.0% ± 0.3% in the control group to 19.7% ± 3.5%, 30.1% ± 2.6%, and 58.2% ± 1.6% at 24, 48, and 72 h of fluzoparib exposure, h respectively (p = 0.014, p < 0.001, p < 0.0001; **Figures 4C, D**). These results indicate that ^131^I-MIBG treatment for 24 h can induce initial DNA damage, while subsequent fluzoparib treatment for 48 h maximizes the accumulation of DNA damage and G2/M arrest. Prolonged fluzoparib exposure to 72 h further promotes apoptotic cell death, as indicated by the increased Sub-G1 population.

To evaluate the dose-response relationship of the combination therapy, PC12-NET cells were treated with ¹³¹I−MIBG for 24 h followed by fluzoparib for 48 h, across a series of dose gradients. The standard combination was defined as ^131^I-MIBG 100μCi/mL and fluzoparib 10μM (1×), with five additional dose gradients established: ^131^I-MIBG 200μCi/mL + fluzoparib 20μM (2×), ^131^I-MIBG 50μCi/mL + fluzoparib 5μM (0.5×), ^131^I-MIBG 25μCi/mL + fluzoparib 2.5μM (0.25×), ^131^I-MIBG 12.5μCi/mL + fluzoparib 1.25μM (0.125×), and a drug-free control (0×). The results revealed that compared to the control group, G2/M phase arrest was observed only under high-dose concentration conditions (1×, and 2×), while the proportion of Sub-G1 phase cells increased progressively with escalating dose concentrations. All groups showed statistically significant differences in Sub−G1 fraction compared to the control (p=0.005, p=0.012, p<0.0001, p=0.011, p=0.0001, respectively) (**Figures 4E, F**). These results indicate that G2/M phase arrest exhibited a threshold effect, where blocking DNA damage repair requires reaching a minimum effective concentration to accumulate irreparable damage. In contrast, the Sub-G1 apoptosis exhibited a linear dose-dependent accumulation effect, consistent with the ability that low-dose beta radiation can directly damage DNA and linearly induce cell apoptosis.

### 3.5 Effects of treatment on cell apoptosis in PC12-NET cells

**Figure 5:**
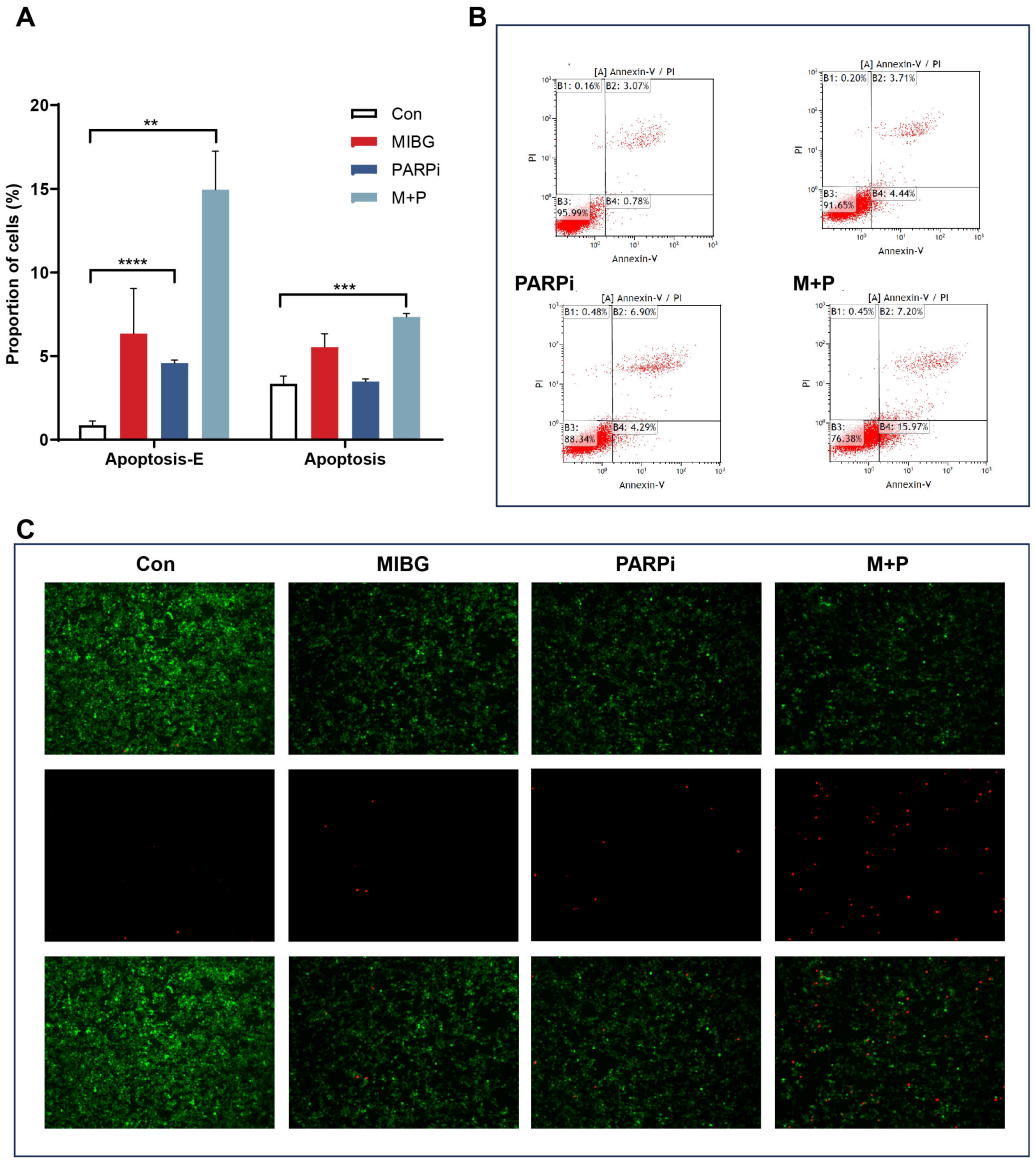
Apoptosis of PC12-NET cells after treatment. **(A)** Quantitative analysis of apoptosis rates in the control and treatment groups. **(B)** Apoptosis status including early and late apoptosis population, and apoptosis rates in control and treatment groups. **(C)** Representative fluorescence images of live cells (green), dead cells (red) and merged images in control and treatment groups. **p < 0.01, ***p < 0.001, ****p < 0.0001.

PC12-NET cells were grouped and treated with monotherapy using100 μCi/mL ^131^I-MIBG (MIBG group) or 10 μM fluzoparib (PARPi group), and combination therapy (M + P group). The combination therapy protocol consisted of 24 h incubation with ¹³¹I−MIBG followed by medium replacement and a further 24 h treatment with fluzoparib. Apoptosis was assessed using flow cytometry and fluorescence microscopy. The results revealed that the combination therapy significantly induced both early and late apoptotic cells than the control group, increasing the proportion of early apoptotic cells from 0.9% ± 0.2% to 15.0% ± 2.3% (p = 0.0036), and the proportion of late apoptotic cells from 3.3% ± 0.3% to 7.3% ± 0.2% (p = 0.0003). Furthermore, fluzoparib monotherapy also induced an increase in the proportion of early apoptotic cells from 0.9% ± 0.2% to 4.6% ± 0.2% (p < 0.0001), while ¹³¹I-MIBG alone did not significantly promote apoptosis (**Figures 5A, B**). Fluorescence microscopy further confirmed that the combination therapy group exhibited a significantly higher number of dead cells compared to the monotherapy groups, signifying that the combination therapy caused more cell apoptosis (**Figure 5C**). The increase in the proportion of early apoptotic cells in the combination therapy was 3.3 times that of fluzoparib monotherapy, which further confirms the presence of a synergistic effect.

### 3.6 Effects of monotherapy and combination therapy in PC12-NET-SDHB cells

**Figure 6:**
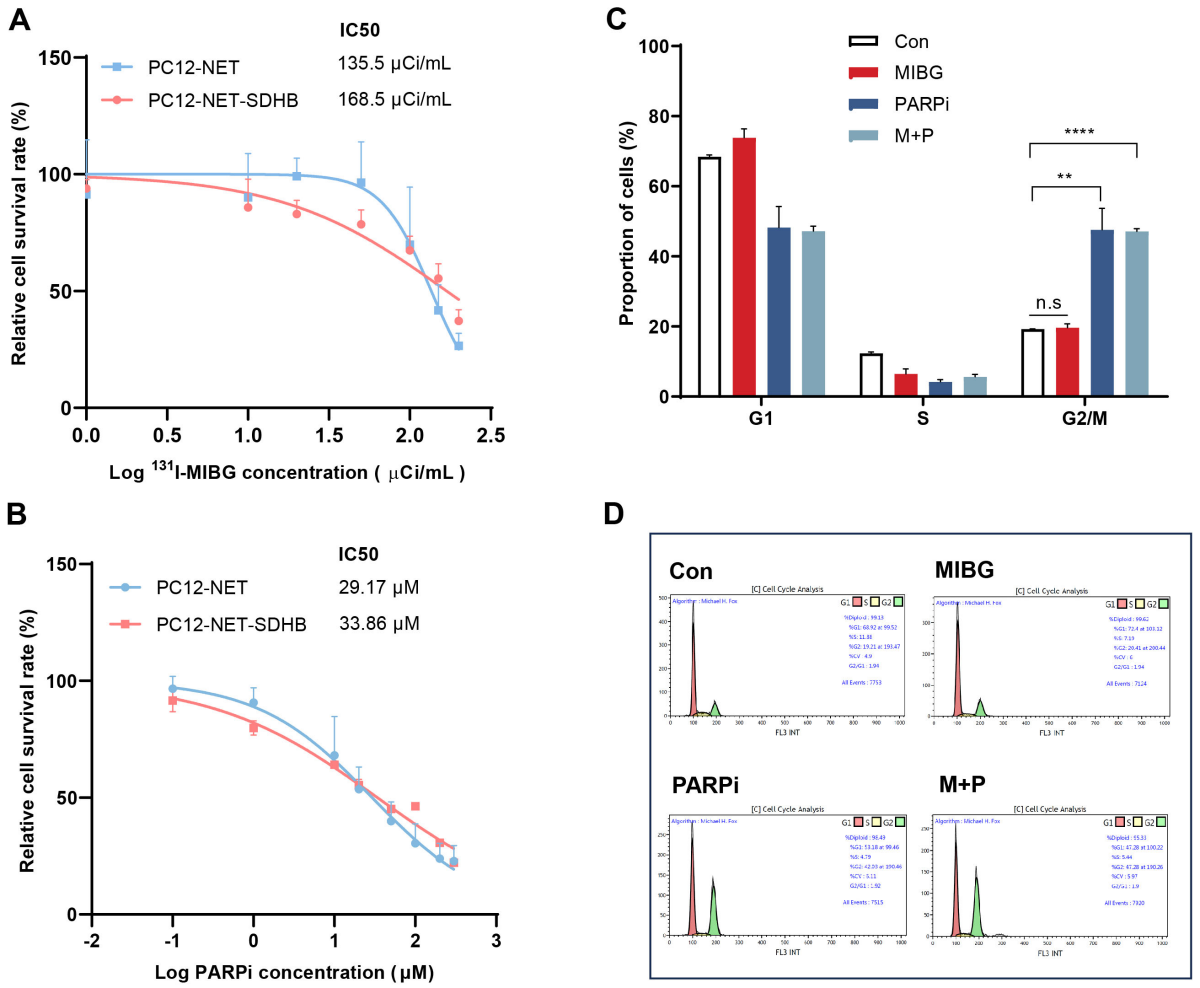
Effects of ¹³¹I-MIBG and fluzoparib on proliferation and cell cycle in PC12-NET-SDHB cells. **(A)** Survival curves of PC12-NET and PC12-NET-SDHB cells following treatment with increasing doses of ^131^I-MIBG. **(B)** Survival curves of PC12-NET and PC12-NET-SDHB cells following treatment with increasing doses of fluzoparib. **(C, D)** Quantitative analysis and distribution of cell cycle in PC12-NET-SDHB cells from the control and treatment groups. **p < 0.01, ****p < 0.0001, n.s: no significance.

To evaluate whether SDHB deficiency influences therapeutic response, PC12-NET-SDHB cells were treated with ^131^I-MIBG monotherapy, fluzoparib monotherapy, or combination therapy, and compare that in PC12-NET cells. The results revealed that ^131^I-MIBG treatment significantly affected cell proliferation dose-dependently for PC12-NET and PC12-NET-SDHB cells, with IC_50_ values of 135.5 and 168.5 μCi/mL, respectively. No significant difference in survival rates was observed between the two cell lines (p = 0.6749; **Figure 6A**). Similarly, fluzoparib monotherapy showed comparable efficacy between the two lines, with IC_50_ values for the inhibition of proliferation activity being 29.17 and 33.68 μM in PC12-NET and PC12-NET-SDHB cells, respectively (p = 0.7151; **Figure 6B**). Cell cycle analysis revealed that fluzoparib monotherapy and combination therapy induced significant G2/M phase arrest in PC12-NET-SDHB cells compared to the control group (p = 0.0013, p < 0.0001,respectively); whereas ^131^I-MIBG monotherapy did not (p = 0.52; **Figures 6C, D**). These results suggest that SDHB deficiency does not significantly alter cellular sensitivity to ^131^I-MIBG or fluzoparib monotherapy. However, at the cell cycle level, fluzoparib monotherapy is sufficient to induce G2/M phase arrest in SDHB-deficient cells, whereas PC12-NET cells require combination therapy to elicit a similar G2/M phase arrest response.

## 4 Discussion

The pathogenesis, progression, and metastatic potential of PPGL are closely associated with specific gene alterations, with SDHB mutations representing the most significant hereditary risk factor for tumor metastasis (22, 23). Treatment options for PPGLs predominantly include localized surgery and palliative care. Currently, high-activity ^131^I-MIBG remains the only radiopharmaceutical approved by the U.S. Food and Drug Administration for the treatment of metastatic PPGLs (24). Among patients with positive MIBG avidity, approximately 78% - 85% derive clinical benefit, with approximately 50% achieving stable disease or partial respond, while 40% reporting symptomatic improvements related to catecholamines excess (25). PARPi exert their antitumor effects by inhibiting PARP-mediated DNA repair pathways and potentially impairing complementary DNA damage response mechanisms, hence enhancing tumor sensitivity to both radiotherapy and chemotherapy (26). In the quest for efficacious therapies for metastatic PPGLs, PARPi combined with temozolomide chemotherapy has been administered to patients with SDHB mutations, with several cases demonstrating partial remission (27). Building on these findings, we propose that combining PARPi with targeted radionuclide therapy may represent a promising strategy to improve clinical outcomes in this challenging patient population.

^131^I-MIBG treatment delivers therapeutic beta particles that induce both single- and double-strand DNA breaks, triggering the activation of cellular DNA repair mechanisms in normal cells. Fluzoparib, in contrast, inhibits the essential enzymes involved in DNA damage repair. In this study, based on initial cell proliferation assays, we employed low doses of ^131^I-MIBG and fluzoparib in a combination treatment strategy in PC12 -NET cells. While each agent alone showed limited efficacy, the combination therapy induced a notable G2/M phase cell cycle arrest, indicating that the extent of DNA damage exceeded the repair capacity of the cells. Moreover, the application of synthetic lethality may alleviate the side effects commonly associated with high-dose radiopharmaceuticals or targeted monotherapies, thereby enhancing therapeutic outcomes when low-dose 131I-MIBG is combined with maintenance-dose PARPi. In apoptosis assays, the combination treatment increased the total apoptotic rate (including early and late apoptosis) to 22.29%, which is nearly tenfold higher than that observed with PARPi monotherapy, thereby highlighting the synergistic pro-apoptotic effect of combining ^131^I-MIBG with fluzoparib in PC12-NET cells.

To evaluate the dose-response relationship of the combined treatment, a series of dose gradients with fixed ratios were administered. The results demonstrated that G2/M phase arrest occurred only at or above the standard dose threshold. Conversely, a significant increase in Sub-G1 phase apoptosis was observed across all dose groups, suggesting two distinct mechanisms of action leading to cell death. The induction of G2/M arrest appears to be dependent on reaching a specific concentration threshold of PARP inhibitor, at which point it effectively impede DNA repair. Notably, even at lower doses, some degree of irreparable DNA damage can occur. This phenomenon may be attributed to the cumulative impact of NET-targeted ^131^I-MIBG treatment, which appears to reduce the threshold for DNA damage tolerance and enhances cellular vulnerability.

In PPGLs harboring SDHB mutations, the enzymatic activity associated with catecholamine metabolism is diminished, resulting in insufficient catecholamine secretion, particularly in early-stage tumors smaller than 3 cm (28). Consequently, MIBG-based diagnosis or assessment is generally not recommended for SDHB-related PPGLs. However, this generalization fails to account for tumor heterogeneity in MIBG uptake and therapeutic response among these tumors. Previous studies have shown that patients with SDHB mutations who exhibit positive ^131^I-MIBG uptake can still benefit from this treatment (29). Case reports have documented instances where patients with SDHB mutations and significant positive MIBG uptake achieved complete remission following treatment with MIBG combined with sunitinib (30). Studies have also demonstrated that a significant loss of SDHB protein expression, primarily resulting from deletions in exon 1, serves as a predictor for negative MIBG uptake (31). Conversely, PPGL lesions in patients with SDHB point mutations may remain high MIBG adivity (32). Our findings demonstrate that SDHB knockdown in PC12-NET cells successfully achieved a significant reduction in SDHB protein expression, while high NET expression was preserved. The derived PC12-NET-SDHB cells respond to ¹³¹I-MIBG treatment comparable to that of PC12-NET cells. This suggests that the efficacy of NET-targeted ¹³¹I-MIBG therapy is not substantially affected by SDHB expression levels, likely due to unaltered drug accumulation. However, an important limitation of this study is the use of an SDHB-knockdown model, which may not fully emulate the biology of hereditary SDHB-mutant tumors. Therefore, caution is warranted when translating these findings to clinical practice. Given the ongoing uncertainty regarding the mechanisms underlying SDHB-related mutations, it may be premature to exclude SDHB mutation status as a criterion for MIBG treatment in patients with PPGLs.

In tumors with SDHB mutations, the accumulation of the metabolite succinate may promote activation of DNA damage repair mechanisms through the PARP1/BER pathway, potentially resulting in increased resistance to conventional therapies (27, 33). Concurrently, the accumulation of succinate in SDHB-mutant cells may lead to impairments in HR repair (31, 34). PARPi target the upregulated PARP/BER repair mechanism in SDHB-mutant cells, thereby enhancing their sensitivity to anticancer therapies (35). This study demonstrated that PARPi monotherapy induced a substantial G2/M cell cycle arrest in SDHB-knockdown cells, with the arrest rate increasing by 3.2-fold (p = 0.0013). This response was notably more pronounced than that observed in PC12-NET cells, consistent with previously reported findings.

However, it is worth noting that the cell proliferation assays failed to reflect the G2/M arrest phenotype observed in cell cycle analysis. This discrepancy aligns with established compensatory pathway mechanisms known to drive resistance in targeted therapies. For instance, MEK inhibitors often exhibit limited efficacy owing to feedback pathway compensation (36). Similarly, SDHB knockdown may trigger adaptive responses that sustain proliferative capacity despite reduced SDHB expression. Alternatively, the observed discrepancy could also arise from assay timing limitations. Future studies should focus on clarifying the fate of cells following cycle arrest and explore the role of metabolic-epigenetic interactions, which may provide deeper insights into therapeutic resistance mechanisms.

### 4.1 Limitations

This study has several limitations. First, it relied solely on the PC12 cell line model and lack validation in animal models or clinical samples. Accordingly, the influence of the *in vivo* metabolic microenvironment could not be assessed. Second, the analysis of apoptotic pathways was not exhaustive, limiting a comprehensive understanding of the treatment’s mechanism of action. Future studies should prioritize elucidating the underlying molecular mechanisms and advance toward clinical trials with long-term follow-up to thoroughly evaluate the therapeutic potential of this combination strategy.

## 5 Conclusions

The combination of ^131^I-MIBG and PARPi demonstrate a notably synergistic therapeutic effect in PC12-NET cells, significantly enhancing treatment response and supporting its potential clinical translation. Furthermore, PC12-NET-SDHB cells retained sensitivity to ^131^I-MIBG comparable to that of PC12-NET cells, while exhibit markedly increased sensitivity to PARPi monotherapy. These results indicates that PARPi may be especially efficacious against SDHB-deficient phonotypes, suggesting a promising therapeutic strategy for improving outcomes in this subset of PPGLs.

## Data Availability

The original contributions presented in the study are included in the article/supplementary material. Further inquiries can be directed to the corresponding author.
